# Coronary artery disease risk reclassification by a new acoustic-based score

**DOI:** 10.1007/s10554-019-01662-1

**Published:** 2019-07-04

**Authors:** S. E. Schmidt, S. Winther, B. S. Larsen, M. H. Groenhoej, L. Nissen, J. Westra, L. Frost, N. R. Holm, H. Mickley, F. H. Steffensen, J. Lambrechtsen, M. S. Nørskov, J. J. Struijk, A. C. P. Diederichsen, M. Boettcher

**Affiliations:** 1 Department of Health Science and Technology, Biomedical Engineering & Informatics, Aalborg University, Fredrik Bajers Vej 7 C1-204, 9220 Aalborg Ø, Denmark; 2 Department of Cardiology, Region Hospital Herning, Herning, Denmark; 3 Department of Cardiology, Odense University Hospital, Odense, Denmark; 4 Department of Cardiology, Aarhus University Hospital, Aarhus, Denmark; 5 Department of Cardiology, Regional Hospital Central Jutland, Silkeborg, Denmark; 6 Department of Cardiology, Lillebaelt Hospital, Vejle, Denmark; 7 Department of Cardiology, Svendborg Hospital, Svendborg, Denmark; 8 Acarix, Lyngby, Denmark

**Keywords:** Stable coronary artery disease, Heart sounds, Non-invasive testing, Reclassification, Cost-effectiveness, Ultrasensitive phonocardiography

## Abstract

To determine the potential of a non-invasive acoustic device (CADScor®System) to reclassify patients with intermediate pre-test probability (PTP) and clinically suspected stable coronary artery disease (CAD) into a low probability group thereby ruling out significant CAD. Audio recordings and clinical data from three studies were collected in a single database. In all studies, patients with a coronary CT angiography indicating CAD were referred to coronary angiography. Audio recordings of heart sounds were processed to construct a CAD-score. PTP was calculated using the updated Diamond-Forrester score and patients were classified according to the current ESC guidelines for stable CAD: low < 15%, intermediate 15–85% and high > 85% PTP. Intermediate PTP patients were re-classified to low probability if the CAD-score was ≤ 20. Of 2245 patients, 212 (9.4%) had significant CAD confirmed by coronary angiography ( ≥ 50% diameter stenosis). The average CAD-score was higher in patients with significant CAD (38.4 ± 13.9) compared to the remaining patients (25.1 ± 13.8; p < 0.001). The reclassification increased the proportion of low PTP patients from 13.6% to 41.8%, reducing the proportion of intermediate PTP patients from 83.4% to 55.2%. Before reclassification 7 (3.1%) low PTP patients had CAD, whereas post-reclassification this number increased to 28 (4.0%) (p = 0.52). The net reclassification index was 0.209. Utilization of a low-cost acoustic device in patients with intermediate PTP could potentially reduce the number of patients referred for further testing, without a significant increase in the false negative rate, and thus improve the cost-effectiveness for patients with suspected stable CAD.

**Electronic supplementary material:**

The online version of this article (10.1007/s10554-019-01662-1) contains supplementary material, which is available to authorized users.

## Introduction

For detection of stable coronary artery disease (CAD), patients undergo risk stratification, non-invasive and invasive testing [1]. However, recent studies have demonstrated that as low as 6–10% of patients referred to non-invasive testing suffer from significant CAD [2–4]. A safe and low-cost rule-out test reducing the number of patients with non-obstructive CAD referred to non-invasive testing could therefore reduce costs and potential risk of complications.

One approach for a simple and efficient tool for ruling out CAD is the automated analysis of heart sounds to identify abnormalities such as weak murmurs related to post stenotic turbulent flow in the coronary arteries [5] and abnormal myocardial vibration patterns [6, 7]. The first report of CAD-related heart sounds originates from the late sixties [8]. Since then a wide range of signal processing algorithms for detection of CAD have been proposed [7, 9–16]. Recently some of these methods have undergone clinical testing [2, 17–20]. One method is the automated stethoscope-like device (CADScor®System, Acarix A/S), which obtains heart sounds from the coronary circulation and myocardium during a 3 min recording period at the 4th left intercostal space. A CAD-score on a scale from 0 to 99 is estimated immediately after the recording using an integrated algorithm performing advanced analysis of the heart sounds in combination with age, gender and blood pressure information. A CAD-score ≤ 20 indicates low probability of CAD and a recent study demonstrated a negative predictive value of 96% in a low to intermediate probability population [2], positioning the device as a potential early rule-out modality before more extensive testing.

In the current study we assessed the potential of the CAD-score algorithm to reclassify patients suspected of stable CAD from intermediate to low likelihood of CAD, to illustrate the rule-out capacity of the CADScor®System.

## Methods

### Study population

Heart sound recordings and patient data from three clinical studies were combined in a database. In short, the Acoustic Data collection for Optimizing CAD-score Algorithm study (AdoptCAD, NCT01564628) included 255 subjects referred for either coronary CT angiography (CTA) or coronary angiography (CAG) [21]. Patients where CTA identified a stenosis were further referred to CAG. A total of 249 patients had a heart sound recording. In the DanRisk 5-year follow-up study (BIO-CAC; NCT02913144), a heart sound recording was obtained in 661 asymptomatic subjects undergoing coronary artery calcium scoring (CACS) [22, 23]. Subjects with a CACS above 400 were offered myocardial scintigraphy and subjects with a CAD-score (algorithm version 2) above 37 (n = 60) were offered CTA. Subjects with a positive CTA or myocardial scintigraphy test were offered CAG (n = 12). In the Dan-NICAD study (NCT02264717), heart sound recordings were successfully obtained in 1563 of 1675 patients with low to intermediate pre-test probability (PTP) referred for CTA with suspicion of CAD [2, 24]. Patients with at least one obstructive stenosis identified at CTA were referred for CAG. All studies were conducted in accordance with the Declaration of Helsinki. Informed consent was obtained from all individual participants included in the studies. The local scientific ethics committees approved the research protocols.

### The CAD-score

A CAD-score was estimated using an offline version of the CAD-score algorithm version 3.1 as embedded in the current CADScor®System. The CAD-score device obtains two recording: first 30 s of pre-test recording to validate the sound quality, next if the pre-test recording passes the algorithm quality control, 150 s are recorded. The heart sound signal is obtained by ultrasensitive phonocardiography using a microphone attached at the 4th intercostal space just to the left of the sternum. The algorithm automatically segments the heart sounds into systolic and diastolic periods [25]. Then the sounds are filtered before eight acoustic features that describe relevant properties of the heart sounds are extracted from the diastolic and systolic periods [2, 6, 26, 27]. These features are combined into an acoustic score using a linear discriminant function. Using logistic regression, the acoustic score is combined with gender, age, and hypertension (systolic blood pressure ≥ 140 mmHg or current treatment with antihypertensive medication) to generate the CAD-score. The CAD-score is scaled so that 90% of patients with CAD have a CAD-score > 20. Hence, a CAD-score value > 20 is categorized as abnormal, for further details see the online supplementary in Winther et al. [2].

The current algorithm version 3.1 was developed and calibrated in a subset including 1201 patients from the current database as described by Winther et al. [2]. Before final implementation of the algorithm in the device, model coefficients for both the linear discriminant analyses and logistic regression and the scaling were fine-tuned in the complete database reported here.

### Reclassification

A simple reclassification scheme was applied to reclassify the probability of CAD in symptomatic patients with suspected CAD from the AdoptCAD and the Dan-NICAD study. PTP was calculated using the updated Diamond-Forrester score [28] according to the ESC guidelines [1]: low < 15%, intermediate 15–85% and high PTP > 85%. Patients in the intermediate PTP group (15–85%) were reclassified using the CAD-score. Patients with an intermediate PTP and a CAD-score ≤ 20 were reclassified to low probability, while patients from the intermediate PTP with a CAD-score > 20 were kept as intermediate probability. Patients with low ( < 15%) or high ( > 85%) PTP were not reclassified.

### Diagnosis

The disease level was divided into three levels: non-CAD, mild-CAD and significant-CAD. Significant-CAD is defined as having a stenosis with at least 50% diameter reduction defined by CAG [29]. Non-CAD is defined as having a CACS at zero and no stenosis identified at CTA. Mild-CAD is having some degree of CAD either CACS higher than zero or having an insignificant stenosis ether by CTA or CAG. Since the diagnostic flow differs from study to study, specific supplementary rules are used in coding of the AdoptCAD and the BIO-CAC study (Supplementary Table 1).

### Statistical analysis

Variables are expressed as mean ( ± standard deviation (SD) or total range). Categorical variables are reported as frequencies (percentages). The unpaired Student t test and ANOVA test were used for comparison between continuous variables. The chi square test was used for comparison between categorical variables. Pearsons correlation was used to analyse correlations between variables. The area under the receiver-operating characteristic (AUC) curve was calculated for continuous variables and in paired designs compared with the method described by DeLong et al. [30] and in unpaired cases with the method of Hanley et al. [31]. The CAD-score was divided as a binary variable with a cut point of 20 and the updated Diamond-Forrester score using a cut point of 15 to calculate sensitivity, specificity, positive and negative predictive values (PPV and NPV), and positive and negative likelihood ratio (PLR and NLR). Performance values are presented with 95% confidence intervals. The post-test probability was calculated using pre-test odds and likelihood ratios by Bayesian statistics. Statistical analyses were performed using Matlab R2017b (MathWorks, US).

Since the current CAD-score algorithm version 3.1 is finetuned in the complete database, the current results could be a result of overfitting of the linear discriminant analysis and logistic regression. To test for overfitting, we did a 50 times repeated tenfold cross-validation test where both the linear discriminant analysis and the logistic regression were re-trained [32].

## Results

In the pooled population, 2473 patients had at least one acoustic heart sound recording. A CAD-score with algorithm version 3.1 could be calculated in 2334 (94%) of the patients, the remaining 139 were excluded from the current analyses. Reasons for not obtaining a CAD-score were arrhythmia (n = 27), algorithm related errors (n = 60), too much noise/too weak heart sounds (n = 34) or missing clinical information such as symptoms or hypertension status (n = 18). Finally, 89 (3.6%) patients were excluded since they could not be assigned a disease level according the diagnostic scheme. The remaining 2245 patients were included in the current analyses.

The mean age of the population was 58.3 ± 8.4 years and included 1185 (52.8%) females and 1060 (47.2%) males (Table 1). The mean PTP for significant CAD according to the updated Diamond-Forrester score was 36.4%. A total of 370 (16.5%) patients had a PTP below 15%, 1824 (81.2%) a PTP between 15 and 85% and 51 (2.3%) had a PTP above 85%. CACS was conducted in 2239 patients (99.7%), 1614 patients (71.9%) underwent CTA and 455 (20.3%) underwent CAG. In total 212 (9.4%) patients had significant-CAD documented by CAG, 44.2% had mild-CAD and 46.4% had non-CAD (Supplementary Table 2).

**Table 1:** Baseline characteristics of included studies

	All	AdoptCAD	Dan-NICAD	BIO-CAC
N	2245	199	1474	572
Sex (female)	1185 (52.8%)	93 (46.7%)	771 (52.3%)	321 (56.1%)
Age	58.3 ± 8.4 [20–86]	61.9 ± 11.0 [20–86]	57.1 ± 8.8 [40–80]	60.1 ± 5.0 [54–66]
BMI	26.9 ± 4.2	27.0 ± 4.3	26.6 ± 4.2	27.5 ± 4.4
Hypertension	1313 (58.5%)	137 (68.8%)	878 (59.6%)	298 (52.1%)
Dyslipidemia	1706 (76%)	159 (79.9%)	1100 (74.6%)	447 (78.1%)
Smoking				
Never	1068 (47.6%)	71 (35.7%)	712 (48.3%)	285 (49.8%)
Former	817 (36.4%)	86 (43.2%)	537 (36.4%)	194 (33.9%)
Active	360 (16.0%)	42 (21.1%)	225 (15.3%)	93 (16.3%)
Diabetes	118 (5.3%)	19 (9.6%)	75 (5.1%)	24 (4.2%)
Family history of CAD				
Yes	680 (30.3%)	0 (0%)	548 (37.2%)	132 (23.1%)
No	1346 (60.0%)	0 (0%)	926 (62.8%)	420 (73.4%)
Undefined	219 (9.8%)	199 (100%)	0 (0%)	20 (3.5%)
Symptoms				
Typical chest pain	490 (21.8%)	85 (42.7%)	399 (27.1%)	6 (1.1%)
Atypical chest pain	608 (27.1%)	81 (40.7%)	505 (34.3%)	22 (3.9%)
Non-specific symptoms	649 (28.9%)	33 (16.6%)	570 (38.7%)	46 (8.0%)
None	498 (22.2%)	0 (0%)	0 (0%)	498 (87.1%)
Updated Diamond-Forrester score	36.4% ± 21.2%	51.1% ± 24.2%	38.6% ± 21.5%	25.9% ± 13.3%
Pre-test probability groups				
< 15%	370 (16.5%)	14 (7.0%)	213 (14.5%)	143 (25.0%)
15–85%	1824 (81.2%)	165 (82.9%)	1230 (83.4%)	429 (75.0%)
> 85%	51 (2.3%)	20 (10.1%)	31 (2.1%)	0 (0%)

### The CAD-score

**Fig. 1:**
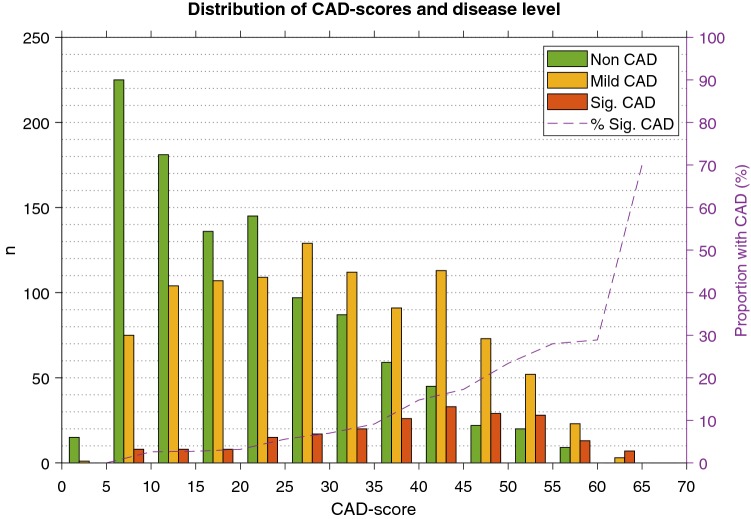
Histogram showing the distribution of CAD-scores in Non-CAD, Mild-CAD and Significant-CAD patients. The dashed line shows the proportion of significant-CAD patients in each bin

The average CAD-score in the pooled population was 26.4 ± 14.3. The average CAD-score was significantly higher in significant-CAD patients 38.4 ± 13.9 versus 25.1 ± 13.8 in the remaining patients (p < 0.001). The distribution of CAD-scores by disease level is shown in Fig. 1. There was a significant stepwise increase in the average CAD-score with increasing severity of disease level (Supplementary Table 3). In 300 patients, one additional recording was obtained after the first recording, the intra-patient correlation between the first and the second CAD-score was r = 0.973 (p < 0.0001).

### Reclassification

**Fig. 2:**
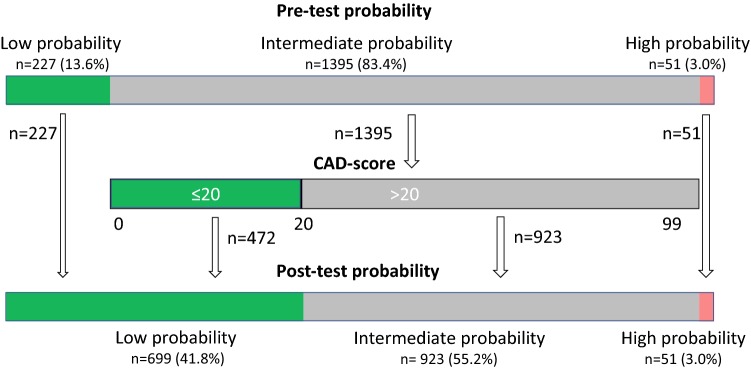
Reclassification results using the propose reclassification scheme where patients with an intermediate PTP is reclassified to low probability in case of CAD-score ≤ 20

Of 1673 patients referred for testing due to suspected CAD (patients from the AdoptCAD and the Dan-NICAD study), 227 (13.6%) patients were classified as having a low likelihood of CAD ( < 15%) according to the PTP estimated by the updated Diamond-Forrester score. Post CAD-score-test this number increased to 699 (41.8%), thus reducing the number of patients classified with intermediate likelihood from 1395 (83.4%) to 923 (55.2%) (Fig. 2). Before testing 7 (3.1%) low PTP patients had significant-CAD, whereas post-reclassification this number increased to 28 (4.0%) (p = 0.52). The net reclassification index was 0.209.

### Diagnostic performance

**Fig. 3:**
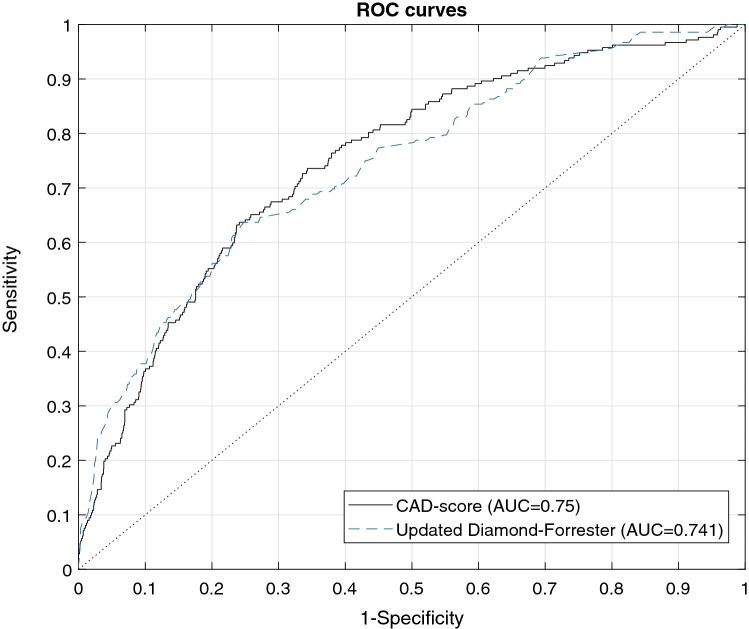
Receiving operating characteristics curve of the CAD-score and the updated Diamond-Forrester score

When separating significant-CAD patients from other patients (non-CAD and mild-CAD) the AUC of the CAD-score was 0.750 (0.710–0.789) (Fig. 3, Table 2). The sensitivity of a CAD-score > 20 was 88.7% (83.6–92.6%) and the specificity of a CAD-score ≤ 20 was 41.5% (39.4–43.7%). The NPV of a CAD-score ≤ 20 was 97.2% (95.9–98.2%) while the PPV of a CAD-score > 20 was 13.7% (11.9–15.6%). The NLR and PLR were 0.27 and 1.52, respectively (Table 2). An increasing CAD-score was associated with a higher risk of having CAD (Fig. 1).

**Table 2:** Diagnostic performance of the CAD-score and the updated Diamond-Forrester score (significant-CAD vs. other)

	All	AdoptCAD	Dan-NICAD	BIO-CAC
CAD-score				
N: Other (Non-CAD and Mild-CAD)	2033	141	1321	571
N: Significant-CAD	212	58	153	1
Prevalence of CAD	9.4%	29.1%	10.4%	0.2%
True negative	844	44	565	235
False negative	24	1	23	0
False positive	1189	97	756	336
True positive	188	57	130	1
AUC	0.750 (0.710–0.789)	0.768 (0.690–0.846)	0.720 (0.673–0.768)	–
Negative predictive value (p = 0.008)	97.2% (95.9–98.2%)	97.8% (88.2–99.9%)	96.1% (94.2–97.5%)	–
Positive predictive value (p < 0.001)	13.7% (11.9–15.6%)	37% (29.4–45.2%)	14.7% (12.4–17.2%)	–
Sensitivity (p = 0.02)	88.7% (83.6–92.6%)	98.3% (90.8–100%)	85% (78.3–90.2%)	–
Specificity (p = 0.03)	41.5% (39.4–43.7%)	31.2% (23.7–39.5%)	42.8% (40.1–45.5%)	41.2% (37.1–45.3%)
Likelihood ratio positive	1.52	1.43	1.49	–
Likelihood ratio negative	0.27	0.06	0.35	–
Updated Diamond-Forrester score				
N: Other (Non-CAD and Mild-CAD)	2033	141	1321	571
N: Significant-CAD	212	58	153	1
Prevalence of CAD	9.4%	29.1%	10.4%	0.2%
True negative	363	14	206	143
False negative	7	0	7	0
False positive	1670	127	1115	428
True positive	205	58	146	1
AUC	0.741 (0.702–0.781)		0.661 (0.612–0.71)	–
Negative predictive value (p = 0.072)	98.1% (96.1–99.2%)	100% (76.8–100%)	96.7% (93.3–98.7%)	–
Positive predictive value (p < 0.001)	10.9% (9.56–12.4%)	31.4% (24.7–38.6%)	11.6% (9.86–13.5%)	–
Sensitivity (p = 0.25)	96.7% (93.3–98.7%)	100% (93.8–100%)	95.4% (90.8–98.1%)	–
Specificity (p < 0.001)	17.9% (16.2–19.6%)	9.93% (5.54–16.1%)	15.6% (13.7–17.7%)	25% (21.5–28.8%)
Likelihood ratio positive	1.18	1.11	1.13	–
Likelihood ratio negative	0.18	0	0.29	–

Negative predictive values, specificity, True Negative, False Negative and Likelihood ratio negative are calculated for CAD-scores ≤ 20 and updated Diamond-Forrester scores < 15%. Positive predictive values, sensitivity, True Positive, False Positive and Likelihood ratio positive are calculated for CAD-scores > 20 and updated Diamond-Forrester scores ≥ 15%

The AUC of the cross-validation, testing for overfitting, was 0.741, which is 0.009 lower than the AUC of the concluding CAD-score.

### Comparison to the updated Diamond-Forrester score

The AUC of the CAD-score was marginally superior to the updated Diamond-Forrester score; 0.750 versus 0.741 (p = 0.64) when separating significant-CAD patients from other patients (Table 2). In patients referred for testing due to suspected CAD (patients from the AdoptCAD and the Dan-NICAD study) the AUC of the CAD-score was 0.749 which was higher (p = 0.01) than the AUC of the updated Diamond-Forrester score 0.703 (p = 0.01). Similar in the Dan-NICAD study the CAD-score performed superior to the updated Diamond-Forrester score with AUCs of 0.720 versus 0.661 (p = 0.01) respectively. In the AdoptCAD study alone the updated Diamond-Forrester score performed comparable to the CAD-score with AUCs of 0.776 versus 0.768 (p = 0.79), respectively. The 15% PTP limit for the updated Diamond-Forrester score resulted in a sensitivity of 96.7% (93.3–98.7%) and a specificity of 17.9% (16.2–19.6%) (Table 2). Combining the CAD-score and the updated Diamond-Forrester score using a linear discriminant function increased the AUC significantly to 0.774 (p = 0.013 versus the CAD-score and p = 0.0002 versus the updated Diamond Forrester score) in the complete database.

### Correlation to disease level and diagnostic performance in sub-groups

**Fig. 4:**
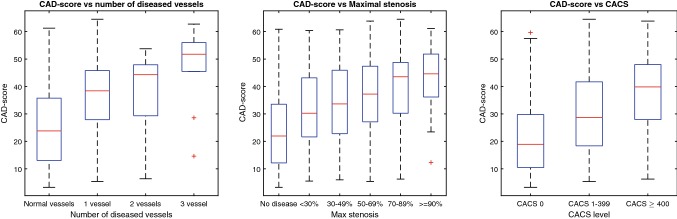
Box plots of CAD-scores dependent on the number of diseased vessels, the maximal stenosis degree according to QCA and the CACS

In patients undergoing CAG a weak correlation (r = 0.23, p < 0.0001) was found between the maximal stenosis degree and the CAD-score and a trend was seen towards an increase in CAD-score with increasing number of diseased vessels (r = 0.22, p < 0.0001) (Fig. 4). The CAD-score correlated with the logarithm of the CACS (r = 0.41, p < 0.0001). The negative predictive value was comparable between males and females, while the sensitivity of the CAD-score was higher in males compared to females.(Table 3). The CAD-score had similar sensitivity in all BMI groups, but there was a trend toward lower specificity with increasing BMI (Table 3). The sensitivity was highest in patients with typical chest pain and non-specific symptoms compared to atypical chest pain. Diabetes reduced the specificity of the CAD-score (Table 3). Only the Dan-NICAD dataset included patients with pathological heart valve disease. In these patients, the sensitivity was increased to 100%, while the specificity was decreased to 23.1% (Table 3).

**Table 3:** Diagnostic performance of the CAD-scores in sub-groups

	n	Prevalence	AUC	Sensitivity	Specificity	NPV	PPV
Gender		p < 0.0001	p = 0.36	p < 0.0001	p < 0.0001	p = 0.69	p < 0.0001
Males	1060	13.9%	0.720 (0.671–0.769)	94.6% (89.6–97.6%)	27.3% (24.4–30.3%)	96.9% (94.0–98.6%)	17.3% (14.8–20.1%)
Females	1185	5.5%	0.688 (0.615–0.761)	75.4% (63.1–85.2%)	53.1% (50.2–56.1%)	97.4% (95.8–98.5%)	8.54% (6.4–11.1%)
Diabetes		p = 0.0001	p = 0.15	p = 0.26	p < 0.0001	p = 0.61	p = 0.0061
Yes	118	19.5%	0.666 (0.535–0.797)	95.7% (78.1–99.9%)	22.1% (14.2–31.8%)	95.5% (77.2–99.9%)	22.9% (15.0–32.6%)
No	2127	8.9%	0.753 (0.712–0.794)	87.8% (82.3–92.1%)	42.5% (40.3–44.7%)	97.3% (95.9–98.3%)	13% (11.2–14.9%)
Symptoms		p < 0.0001	p = 0.0066	p = 0.012	p = 0.66	p = 0.076	p < 0.0001
Typical chest pain	490	19.6%	0.795 (0.739–0.851)	92.7% (85.6–97.0%)	43.7% (38.7–48.7%)	96.1% (92.1–98.4%)	28.6% (23.7–34.0%)
Atypical chest pain	608	9.9%	0.691 (0.614–0.768)	78.3% (65.8–87.9%)	41.2% (37.1–45.5%)	94.6% (90.9–97.1%)	12.7% (9.5–16.6%)
Non-specific symptoms	649	8.5%	0.746 (0.669–0.823)	92.7% (82.4–98.0%)	40.9% (36.9–45.0%)	98.4% (95.9–99.6%)	12.7% (9.6–16.3%)
BMI		p = 0.53	p = 0.013	p = 0.73	p < 0.0001	p = 0.66	p = 0.027
< 20	70	10.0%	0.823 (0.628–1.00)	100% (59.0–100%)	58.7% (45.6–71.0%)	100% (90.5–100%)	21.2% (9.0–38.9%)
20 and < 25	724	10.6%	0.791 (0.729–0.852)	89.6% (80.6–95.4%)	49.5% (45.5–53.4%)	97.6% (95.3–98.9%)	17.4% (13.8–21.5%)
25 and < 30	962	8.5%	0.706 (0.641–0.771)	86.6% (77.3–93.1%)	39.7% (36.4–43.0%)	96.9% (94.6–98.5%)	11.8% (9.3–14.6%)
30	483	9.3%	0.750 (0.666–0.835)	88.9% (75.9–96.3%)	31.5% (27.2–36.1%)	96.5% (92.0–98.9%)	11.8% (8.5–15.7%)
Heart valve disease*		p = 0.81	p = 0.53	p = 0.37	p = 0.006	p = 0.56	p = 0.90
Yes	58	10.3%	0.686 (0.440–0.930)	100% (54.1–100%)	23.1% (12.5–36.8%)	100% (73.5–100%)	13.0% (4.9–26.3%)
No	2187	9.4%	0.750 (0.710–0.790)	88.3% (83.2–92.4%)	42.0% (39.8–44.2%)	97.2% (95.9–98.2%)	13.7% (11.9–15.6%)

Negative predictive values (NPV) and specificity are calculated for CAD-scores ≤ 20. Positive predictive values (PPV) and sensitivity are calculated for CAD-scores > 20

*Only the Dan-NICAD included subjects with heart valve disease

## Discussion

Recent findings of low diagnostic yield at non-invasive testing calls for a more rational approach to avoid unnecessary testing, providing both clinical and economic advantages. In this study we analysed the rule-out potential of a new CAD-score utilized before non-invasive testing of patients with suspected stable CAD. We found that the CAD-score enabled a significant and safe reclassification of patients, which could reduce the need for more expensive testing in patients presenting with chest pain.

### The CADScor®System as a rule-out device

According to the current ESC guideline patients with intermediate PTP (15–85%) should undergo non-invasive testing [1]. In patients referred for testing due a suspicion of CAD we reclassified patients from the intermediate PTP group into the low probability group for negative CAD-scores. Thereby 699 (41.8%) patients could potentially avoid further costly testing, which is more than three times as many as if only the Diamond-Forrester score was used for rule-out (227 patients, 13.6%). Of notice, the 2016 NICE guidelines mention the CAD-score as a potential clinically relevant prediction model [33]. The proposed procedure was associated with a minor and insignificant increase in the proportion of significant-CAD patients in the low probability group from 3.1% to 4.0%.

A positive CAD-score ( > 20) resulted in a sensitivity of 88.7% which in the present low prevalence population (9.4% CAD) leads to a NPV at 97.2%. Thereby the probability of having significant-CAD was 2.8% for patients with a negative CAD-score ( ≤ 20). This probability is much lower than the 15% PTP threshold defined by the ESC guidelines for stable CAD that states that it is safe to assume that patients with a PTP below 15% have no significant CAD and no further testing is recommended. This suggests that the CAD-score safely rules-out CAD in the low and intermediate PTP population. The proposed use of the CADScor®System is as a first-line test before other non-invasive testing. This is reflected in the Dan-NICAD population which had an average PTP at 38.6%, where the CAD-score had a significantly higher AUC than the Diamond-Forrester score.

### The CAD-score in sub-groups

Investigating the effect of risk factors potentially interfering with the CAD-score result, such as high BMI, diabetes or heart valve disease resulted in similar or increased sensitivity of the CAD-score in sub-group analyses, and in lower specificity, see Table 3. This indicates that the rule-out efficacy is lower in these sub-groups, but the rule-out safety is the same as in the remaining population. As in other risk models including gender, the sensitivity was lower in females compared to males. Despite this, the CAD-score had comparable rule-out safety in males and females, with similar negative predictive values.

### Study limitations

The current study is a retrospective analysis of pooled data from existing cohorts and might therefore not capture all aspects of the clinical workflow. The database included a group of asymptomatic subjects from a screening study and it included a group of patients referred for CAG. Neither of these subjects are typical representatives for patients referred for non-invasive testing. However, the baseline characteristics such as age, gender and PTP of the pooled data corresponded well to the characteristics of the Dan-NICAD study which included only patients referred for non-invasive testing. The conclusion of the current study is limited to low to intermedia risk patients since the number of high-risk patients (updated Diamond Forrester score > 85%) was very low in the current study. The CAD-score algorithm described in the current paper is finetuned in the complete database before implementation in the CAD-score device. This induces the risk of overfitting the algorithm to the data, however the cross-validation of the algorithm showed only a small decrease in AUC of 0.009 thereby the degree of overfitting can be considered unimportant for the overall results. As recommended in the current ESC guidelines, the updates Diamond-Forrester score was used for PTP estimation. Other risk assessment models like the CAD-consortium scores [34] or PROMISE Minimal-Risk Tool [35] estimate lower risk levels which might alter interaction between PTP and the CAD-score. To further understand the interaction between long term in risk and CAD-scores future studies should include long term follow up data.

## Conclusion

In the current study, we simulated use of the CAD-score to rule-out CAD in patients with intermediate PTP and suggest that the method potentially can reduce the number of patients who should be referred for non-invasive testing, without a significant increase in the false negative rate. If these finding can be replicated in prospective studies, the use of the CAD-score could significantly alter the current practise of early rule-out of stable CAD providing important clinical and economic advantages.

## Electronic supplementary material

Below is the link to the electronic supplementary material.

Supplementary file1 (DOCX 16 kb)
