# Supplementary material for: Coronary artery disease risk reclassification by a new acoustic-based score
Source: Int J Cardiovasc Imaging. 2019 Jul 4;35(11):2019–28. doi: 10.1007/s10554-019-01662-1 (PMC6805823; doi:10.1007/s10554-019-01662-1)
Supplement: Supplementary file 1 — Supplementary file1 (DOCX 16 kb) [file 10554_2019_1662_MOESM1_ESM.docx]

# Online supplementary

Table 1s. Rules for coding of the three types of disease level.

| **CAD classification** | **General rules** | **Supplementary study specific rules (or)** | | |
| --- | --- | --- | --- | --- |
|  | *All studies* | *Dan-NICAD* | *AdoptCAD* | *BIO-CAC* |
| Non-CAD | CACS=0 & CTA=Normal | - | CACS=0 & CAG<30% & No CTA | CACS=0 |
| Mild-CAD | CACS>0 & CTA Normal & no CAG  or  CTA=Mild-moderate & no CAG  or  2D-CAG (<50%) & (CTA not normal or CACS>0) | - | - | CACS>0 & CACS<400 & No CTA & No SPECT & No CAG  or  (SPECT Normal & CACS>0) |
| Significant-CAD | 2D-CAG >=50% | - | - | - |

Table 2s. Diagnostic modalities and results

|  | **All** | **AdoptCAD** | **Dan-NICAD** | **BIO-CAC** |
| --- | --- | --- | --- | --- |
| CACS | 2239 (99.7%) | 195 (98.0%) | 1472 (99.9%) | 572 (100%) |
| *Level* |  |  |  |  |
| 0 | 1096 (48.8%) | 70 (35.2%) | 763 (51.8%) | 263 (46.0%) |
| 1-399 | 927 (41.3%) | 78 (39.2%) | 572 (38.8%) | 277 (48.4%) |
| ≥400 | 216 (9.6%) | 47 (23.6%) | 137 (9.3%) | 32 (5.6%) |
| CTA | 1614 (71.9%) | 89 (44.7%) | 1473 (99.9%) | 52 (9.09%) |
| *Conclusion* |  |  |  |  |
| Non-CAD | 822 (36.6%) | 63 (31.7%) | 716 (48.6%) | 43 (7.5%) |
| Mild-moderate | 451 (20.1%) | 16 (8.0%) | 432 (29.3%) | 3 (0.5%) |
| Severe | 341 (15.2%) | 10 (5.0%) | 325 (22.0%) | 6 (1.1%) |
| CAG | 455 (20.3%) | 120 (60.3%) | 326 (22.1%) | 9 (1.6%) |
| *Conclusion* |  |  |  |  |
| No stenosis | 117 (5.2%) | 42 (21.1%) | 74 (5.0%) | 1 (0.2%) |
| Stenosis <50 % diameter reduction | 126 (5.6%) | 20 (10.1%) | 99 (6.7%) | 7 (1.2%) |
| Stenosis ≥50 % diameter reduction | 212 (9.4%) | 58 (29.1%) | 153 (10.4%) | 1 (0.2%) |
| *Concluding diagnosis* |  |  |  |  |
| Significant-CAD | 212 (9.4%) | 58 (29.1%) | 153 (10.4%) | 1 (0.2%) |
| Mild-CAD | 992 (44.2%) | 79 (39.7%) | 605 (41.0%) | 308 (53.8%) |
| Non-CAD | 1041 (46.4%) | 62 (31.2%) | 716 (48.6%) | 263 (46.0%) |

Table 3s CAD-scores and CAD levels

| CAD-score values | All | Significant-CAD | Mild-CAD | Non-CAD |
| --- | --- | --- | --- | --- |
| All (p<0.001) | 26.4±14.3 | 38.4±13.9 | 29.5±13.8 | 20.9±12.5 |
| AdoptCAD (p<0.001) | 34.5±16.7 | 45.5±12.4 | 36.3±15.1 | 22±13.7 |
| Dan-NICAD (p<0.001) | 25.7±14.0 | 35.7±13.6 | 29.1±13.6 | 20.8±12.5 |
| BIO-CAC (p<0.001) | 25.1±13.4 | 29±0 | 28.6±13.3 | 21±12.2 |
